# Effects of Ground Transport in Kemp’s Ridley (*Lepidochelys kempii*) and Loggerhead (*Caretta caretta*) Turtles

**DOI:** 10.1093/iob/obaa012

**Published:** 2020-05-19

**Authors:** K E Hunt, C Merigo, E A Burgess, C Loren Buck, D Davis, A Kennedy, L Lory, J Wocial, K McNally, C Innis

**Affiliations:** 1 Smithsonian-Mason School of Conservation & George Mason University Department of Biology, Front Royal, VA, 22630, USA; 2 Rescue and Rehabilitation Department, New England Aquarium, Boston, MA 02110, USA; 3 Anderson Cabot Center for Ocean Life, New England Aquarium, Boston, MA 02110, USA; 4 Department of Biological Sciences, Northern Arizona University, Flagstaff, AZ 86011, USA; 5 Idexx Laboratories, 3 Centennial Drive, North Grafton, MA 01536, USA; 6 Animal Health Department, New England Aquarium, Boston, MA 02110, USA

## Abstract

Many juvenile Kemp’s ridley (*Lepidochelys kempii*) and loggerhead (*Caretta caretta*) turtles strand during fall on the beaches of Cape Cod (MA, USA), with total stranding numbers sometimes exceeding 300 turtles per year. Once rehabilitated, turtles must be released at beaches with appropriate water temperatures, often requiring transportation to southeastern coastal states of the USA. These transportation events (transports) may approach or exceed 24 h in duration. Kemp’s ridley turtles are known to exhibit an adrenal stress response during such transports, but the effect of transport duration has been unclear, and no other sea turtle species has been investigated. To assess whether transport duration and/or species affects physiological reactions to transport, we studied pre- and post-transport physiological measures in Kemp’s ridley and loggerhead turtles transported by ground for <6, ∼12, ∼18, or ∼24 h, comparing with matched “control events” in which turtles were studied without transport. Blood samples were analyzed for four stress-associated measures (corticosterone, glucose, total white blood cell [WBC] count, and heterophil/lymphocyte ratio [H/L]) and nine measures of clinical status (pH, pO_2_, pCO_2_, HCO_3_, sodium, potassium, ionized calcium, lactate, and hematocrit). In both species, stress-associated measures elevated significantly during transport, while handling without transport had no significant effects. Loggerheads exhibited a greater stress response than Kemp’s ridleys across all transport durations. These results indicate that sea turtles do react physiologically to ground transport; therefore, minimizing transport time and streamlining transport logistics (where feasible) may help ensure release of rehabilitated turtles to sea in the best possible condition. Nonetheless, both species remained in good clinical condition even after 24 h transport, indicating that current transport protocols are generally safe for sea turtles from a clinical perspective.

## Synopsis

(Brazilian Portuguese translation) Muitos juvenis de tartaruga-de-kemp (*Lepidochelys kempii*) e de tartaruga-cabeçuda (*Caretta caretta)* encalham nas praias de Cape Cod (MA, EUA) durante o outono, frequentemente o número de encalhamentos pode ultrapassar 300 tartarugas por ano. Uma vez reabilitadas, as tartarugas precisam ser soltas em praias que apresentem água em temperatura apropriada, o que muitas vezes requer o transporte para a costa sudeste dos EUA. Esse transporte (transports) pode durar 24 h ou mais. As tartarugas-de-kemp exibem ativação da adrenal em resposta ao estresse de transporte, porém os efeitos do tempo de transporte permanencem desconhecidos, e nenhuma outra espécie de tartaruga marinha foi investigada nesse aspecto. Para entender os efeitos da duração do transporte e/ou espécie, estudamos medidas fisiológicas pré- e pós-transporte em tartarugas-de-kemp e tartarugas-cabeçudas transportadas por solo durante <6, ∼12, ∼18, ou ∼24 h, comparando com eventos controle (control events) nos quais as tartarugas estudadas não foram transportadas. Amostras de sangue foram analisadas para quatro medidas associadas ao estresse (corticosterona, glicose, contagem total de células [WBC] brancas, e razão heterófilo/linfócito [H/L]) e nove medidas de condição clínica (pH, pO_2_, pCO_2_, HCO_3_, sódio, potássio, cálcio ionizado, lactato, e hematócrito). Nas duas espécies, as medidas associadas ao estresse foram significativamente elevadas durante o transporte, enquanto a manipulação sem transporte não apresentou efeitos significativos. As tartarugas-cabeçuda exibiram maior resposta ao estresse comparada com as tartarugas-de-kemp em todas as durações de transporte. Esses resultados indicam que essas tartarugas marinhas reagem fisiologicamente ao transporte por solo. Portanto, minimizar o tempo e otimizar a logística de transporte (quando possivel) poderia assegurar que tartarugas reabilitadas tenham a melhor condição possível para a soltura. Não obstante, indivíduos das duas espécies permaneceram em boas condições clínicas mesmo após 24 h de transporte, indicando que da perspectiva clínica, os protocolos de transporte atuais são seguros para as tartarugas marinhas.

## Introduction

Over the past decade, increasing numbers of sea turtles have stranded on the shores of Cape Cod, MA, USA, during the months of October–December every year (Allman 1998; Gerle et al. 2000; Still et al. 2005; Rockwell et al. 2017; Innis et al. 2019; National Oceanographic and Atmospheric Administration [NOAA] 2019). More than 100 stranded turtles on average are currently found on Cape Cod each autumn, with strandings in some years exceeding 300 live-stranded individuals (Innis and Merigo 2014; Griffin et al. 2019). The majority of these stranded turtles are juvenile Kemp’s ridley sea turtles (*Lepidochelys kempii*, “Kemp’s ridleys”), a critically endangered species (International Union for the Conservation of Nature [IUCN] 2019; United States Fish and Wildlife Service [USFWS] 2019), with loggerhead turtles (*Caretta caretta*, “loggerheads”), a vulnerable species, the next most common. The stranded Kemp’s ridleys and loggerheads are usually found “cold-stunned,” a hypothermia-like state entailing low body temperature and related clinical abnormalities that include electrolyte derangements, dehydration, acidosis, pneumonia, sepsis, and injuries (Innis et al. 2009; Innis and Staggs 2017). Most admitted turtles are rehabilitated successfully (Wyneken et al. 2006; Innis and Merigo 2014) and are usually ready for release back to sea by approximately March of the following year. However, local water temperatures along the Massachusetts coast are still too low at that time for safe release of these ectothermic species. Therefore, every spring, numerous sea turtles are transported south along the eastern seaboard of the USA in order to release them at beaches with appropriate water temperatures (Hunt et al. 2016a, 2019; NOAA 2019).

These transportation events (“transports”) typically occur by ground in small trucks or vans, with the turtles out of water in individual padded crates or boxes. Transport durations may range from <6 h (to local New England beaches) to >24 h (to Florida beaches), depending on calendar month, local water conditions at potential release sites, and other logistical constraints (Hunt et al. 2016a, 2019). It is therefore of interest to determine the degree of physiological stress that sea turtles might experience during transport events of various durations, as well as to evaluate possible effects of species differences, so as to identify optimal methodology for transporting sea turtles by ground.

Some stress is inevitable when transporting wildlife (Dickens et al. 2010). Ground transport invariably exposes animals to numerous novel stimuli including confinement in a small and unfamiliar container, noise, vibration, unpredictable acceleration, lack of food, and, for sea turtles, removal from the aquatic environment (Grandin 2007; Hunt et al. 2016a, 2019). In vertebrates, such stimuli typically elicit a classic physiological stress response characterized by a marked increase in adrenal catecholamines within seconds of initial disturbance, followed by a more gradual and prolonged elevation in adrenal glucocorticoids (corticosterone in turtles) (Gregory et al. 1996; Milton and Lutz 2002; Grandin 2007; Knowles and Warriss 2007). In ectotherms such as turtles, the elevation of glucocorticoids may last hours (e.g., Gregory et al. 1996; Gregory and Schmid 2001; Jessop and Hamann 2005). Generally, glucocorticoids increase fuel availability (e.g., gluconeogenesis and lipolysis), alter behavior (e.g., increased foraging, abandonment of familiar areas, and increased spatial movement), and suppress nonessential processes (e.g., growth, reproduction; Sapolsky et al. 2000; Romero and Wingfield 2015). Immune function is affected as well, with glucocorticoids causing shifts in distribution of white blood cells (WBCs), detectable via increased heterophil/lymphocyte ratio [H/L ratio] and sometimes increased total WBC count (Martin 2009; Romero and Wingfield 2015). Although these changes are adaptive in the short term, redirecting available energy toward coping with the stressor and normalizing metabolic responses (Romero and Wingfield 2015), prolonged or frequent activation of the stress response has pervasive negative effects that can include suppression of growth, impaired immune response, slowed reactions to subsequent novel stressors, delays in wound-healing time, inhibition of current reproductive efforts, and long-term alterations in adult behavior and future reproduction (reviewed in Romero and Wingfield 2015). In wildlife that are released immediately after transportation, transport-related stress increases chance of abandonment of the release site, vulnerability to predators and disease, and risk of mortality (Jenni et al. 2015; Romero and Wingfield 2015). Although long-term impacts of stress have not been assessed in chelonians, these patterns are observed consistently in other vertebrates (Romero and Wingfield 2015). Therefore, wildlife transport practices are typically designed to minimize and/or ameliorate stress where feasible (Grandin 2007; Teixeira et al. 2007; Dickens et al. 2010; Jenni et al. 2015; Hunt et al. 2019). Such an approach requires information on the degree to which transportation affects physiological stress in a given taxon.

In prior studies, we confirmed that Kemp’s ridley turtles do experience an adrenal stress response during ground transports of 13 and 26 h duration, with pronounced and significant elevations in plasma corticosterone, plasma glucose, WBC, and H/L ratio immediately post-transport when compared with pre-transport (Hunt et al. 2016a, 2019). Controlled experiments clarified that these changes are primarily due to transportation, with only minor effects attributable to handling, food restriction, or time of day (Hunt et al. 2016a). However, no information on transport stress exists for loggerheads, the second most common species found in the annual Cape Cod mass stranding events. Loggerheads are a larger species with greater body mass, and might react differently to out-of-water transport events. Even for Kemp’s ridleys, a relatively small sea turtle species that has been best studied regarding transport stress, no information exists on advisable transport duration. Without information on the potential effects of duration and species differences, it is difficult to determine best practices for ground transportation of sea turtles.

In this study, we sought to fill this information gap by evaluating physiological stress responses of juvenile Kemp’s ridley and loggerhead turtles transported for four different durations (<6, ∼12, ∼18, and ∼24 h), representing typical transport durations to four commonly used release sites along the US eastern seaboard. Our study design relied on opportunistic sampling of turtles already scheduled for transport. We compared pre- and post-transport measures of four “stress-associated” variables identified in prior studies (corticosterone, glucose, WBC, and H/L ratio, all previously shown to elevate during transport of Kemp’s ridleys; Hunt et al. 2016a, 2019). We also assessed nine “clinical health measures” (pH, pO_2_, pCO_2_, HCO_3_ [bicarbonate], sodium, potassium, calcium, lactate, and hematocrit) that are commonly used to monitor clinical status, following Hunt et al. (2012, 2016a, 2016b, 2019). Ground transport events were compared with “control events” in which turtles were handled and sampled twice (with food withheld, but without transport) allowing us to control for potential effects of repeated handling, food restriction, and time of day. Our primary questions were (1) does duration of transport (<6, ∼12, ∼18, and ∼24 h) affect degree of physiological stress response in sea turtles? (2) Are there species differences between Kemp’s ridleys versus loggerheads after the same durations of transport, and, if so, which species is more sensitive to transport stress? And (3) are our current transport protocols generally safe for sea turtles, at all tested durations and for both species?

## Materials and methods

### Study subjects

All turtles in this study had stranded in a cold-stunned state along the shore of Cape Cod Bay during October–December of 2011–2017, and were admitted to the New England Aquarium (NEAq) rehabilitation clinic in Quincy, MA, USA. Transport of any individual turtle occurred the spring or summer after that turtle’s initial date of stranding, with all turtles transported <10 months after stranding. Kemp’s ridley turtles in this study were estimated to be 2–3 years of age (B. Higgins, National Marine Fisheries Service, personal communication; Avens and Snover 2013), and loggerhead turtles 5–10 years of age (Casale et al. 2009, 2011), based on carapace length at the time of stranding. Loggerheads were generally much heavier and larger than Kemp’s ridleys (see the “Results” section).

Our clinical treatment of cold-stunned turtles has been fully described elsewhere (Wyneken et al. 2006; Innis and Staggs 2017). Briefly, turtles were gradually rewarmed during their first week of hospitalization while medical problems were treated. Once swimming well, turtles were placed in large tanks with other similar-sized turtles, with individualized veterinary treatment continued as necessary. Turtles were subsequently maintained in saltwater tanks at ∼26°C (the average of the appropriate temperature range for these species; Renaud 1995; Coles and Musick 2000; Hawkes et al. 2007) as described in Hunt et al. (2012), with daily health monitoring and twice-daily feeding (herring and squid offered individually to each turtle).

Behavior, corticosterone, and other physiological measures of cold-stunned Kemp’s ridley and loggerhead turtles return to presumed-normal levels after approximately 2 months in the NEAq rehabilitation clinic (e.g., Hunt et al. 2012); turtles in this study had all been housed in the NEAq clinic for 6–8 months before participating in any transport event or control event. Prior to inclusion in a given transport event or control event, all turtles were judged by veterinary staff to be clinically stable and in good condition based on serial physical examinations, hematologic and plasma biochemical analysis, and radiographic evaluation.

The NEAq sea turtle rehabilitation program and associated research were conducted with the authorization of the US Department of the Interior Fish and Wildlife Service (permit number TE-697823), in compliance with guidelines of the Animal Care and Use Committees of the NEAq (IACUC protocols 2012-03, 2015-10, and 2018-06) and Northern Arizona University (IACUC protocol 16-007). Turtle transports and beach releases were performed in compliance with all applicable local, state, and federal regulations and guidelines.

### Sampling, handling, and transportation

#### Selection of event durations

Our experimental design involved opportunistic study of Kemp’s ridleys and loggerheads that were scheduled for transport to four frequently used release locations: local Massachusetts beaches (<6 h transport, typically 3–6 h), the Chesapeake Bay area (Virginia and Maryland; ∼12 h transport), the coast of Georgia (∼18 h transport), or the coast of northern Florida (∼24 h transport). Transports were thus grouped into four duration bins of <6, ∼12, ∼18, and ∼24 h, with actual transport durations expected to fall within ±2 h of the target transport time. Unpredictable traffic conditions and weather sometimes cause a transport to deviate by ∼1–2 h from the planned transport time. Our planned sample size, based on variation observed in pilot data, was *n *=* *8 turtles per species per transport duration bin.

Transport events were compared with data from matched control events during which turtles were handled and sampled twice, but with no transport (details below). Control events occurred 2–3 weeks prior to the matched transport event, with the control event matching the scheduled transport event for estimated duration, time of day, species, and number of turtles. Where possible, the same turtles were used for a matched control event and a transport event (same duration), but it was not always possible to study the same turtles for the control event. No turtle was studied for multiple events of the same type and duration, i.e., pseudoreplication did not occur.

#### Pre-event sampling

At the initiation of each control event or transport event, turtles were removed from their pools at the NEAq sea turtle rehabilitation clinic in Quincy, MA, USA, and a 2–3 mL heparinized blood sample (“pre” sample) was immediately collected from the external jugular vein as previously described (Innis et al. 2007, 2009), followed by measurements of cloacal temperature, heart rate, and respiratory rate. Since handling and sampling can themselves cause stress responses (Romero and Reed 2005; Hunt et al. 2016a), blood sampling time (“bleed time”) and total handling time were recorded (beginning from the moment that the capture net first entered the turtle’s pool, and ending at the completion of blood sampling). Bleed time was generally <5 min and handling time <15 min (see details in the “Results” section); our prior studies indicate that these sampling and handling protocols have only minimal effects on the physiological measures investigated here (Hunt et al. 2016a). If the turtle had not been weighed and measured within the previous 7 days, its body mass (to nearest 0.01 kg), straight carapace length (SCL; notch-to-tip, to nearest 0.1 cm), and straight carapace width (SCW; at widest point, to nearest 0.1 cm) were measured after all other clinical data were obtained. Once handling was complete, turtles were either returned to their tanks (control events) with food withheld, or were prepared for ground transport (transport events).

#### Transportation protocol

After sampling and examination, turtles scheduled for transport were placed into individual padded crates with air holes, given subcutaneous fluid therapy (lactated Ringer’s solution, 10 mL/kg), and coated lightly with water-soluble lubricant to reduce dehydration, following the NEAq clinic’s routine transport protocols. Crates were closed and loaded into the rear cargo area of large sport utility vehicles with ambient temperature set to 26°C, the same temperature as the NEAq holding tanks. Vehicle temperature was monitored continuously with digital remote temperature probes placed in the rear cargo area near the turtles. For <6 and ∼12 h transports, pre-transport sampling occurred in the morning, with post-transport sampling occurring later on the same day, i.e., these transport durations did not include an overnight phase. The ∼18 and ∼24 h transports were overnight events, with pre-transport sampling occurring in the morning (∼24 h transports) or afternoon (∼18 h transports), driving through the night, and post-transport sampling occurring the following morning. In all transport events, efforts were made to minimize potential disturbance to turtles, streamline logistics, and minimize driving durations. Brief stops (∼15 min) for refueling and driver-change occurred approximately every 3–4 h.

#### Post-event sampling

For control events, turtles were removed from tanks and re-sampled (“post” sample), as described above, after the designated event duration (<6, ∼12, ∼18, and ∼24 h). For transport events, upon arrival at the destination, turtles were unloaded from vehicles and carried (still in closed crates) to a shaded area for sampling. The transport crate was then opened, with the moment of opening of the crate designated as “time zero” for measurements of bleed time. A second heparinized blood sample was immediately taken (“post” sample) and a second clinical examination performed using the methods described above, with results compared with pre-transport samples and to control events.

### Blood analyses: i-STATs, complete blood counts, and corticosterone assays

#### Sample analysis

Our analytical protocols are described in detail in Hunt et al. (2019). In short, whole-blood samples were divided into three portions, with 0.20 mL used for point-of-care i-STAT clinical chemistry analyses of electrolytes, glucose, lactate, blood gases, and pH, and ∼0.5–1.0 mL shipped on ice to an outside laboratory for a complete blood count (CBC; IDEXX Reference Laboratories, North Grafton, MA, USA). Remaining blood was refrigerated until all turtles had been sampled (∼45–60 min) and then centrifuged at 1500 *g* for 5 min, with plasma pipetted to a vapor-proof cryovial and shipped on dry ice to the endocrine laboratory of the Buck Laboratory, Northern Arizona University, Flagstaff, AZ, USA, for corticosterone assay (details below).

#### Clinical chemistry (i-STAT analyses)

Immediately upon collection of the sample, whole blood was loaded into both an i-STAT CG4+ Test Cartridge and an i-STAT CG8+ Test Cartridge, which were analyzed using a portable battery-powered handheld point-of-care analyzer, VetScan i-STAT^®^ 1 Analyzer Model 300A (Abbott Point of Care, Princeton, NJ, USA). Two i-STAT machines were used; each turtle’s two samples were always analyzed by the same i-STAT machine. Generally, data appeared comparable across machines (data not shown).

The CG4+ cartridges measured plasma pH, pO_2_, pCO_2_, HCO_3_ (bicarbonate), and lactate. The CG8+ cartridges measured sodium, potassium, ionized calcium (iCa), and glucose, and also produced backup data on pH, pO_2_, pCO_2_, and HCO_3_. Data for pH, pO_2_, pCO_2_, and HCO_3_ were taken from the first cartridge analyzed, which in almost all cases was the CG4+ cartridge. In three cases (a 12 h post-transport sample, an 18 h post-control sample, and a 24 h pre-transport sample, all from Kemp’s ridleys), the CG4+ cartridge initially failed and hence the CG8+ cartridge was then analyzed first while a new CG4+ cartridge was prepared, in order to minimize delays in obtaining blood gas measurements. The pH, pO_2_, and pCO_2_ data were all temperature-corrected for that turtle’s cloacal temperature, and iCa data were pH-corrected using previously described formulas (Keller et al. 2012). HCO_3_ concentration was calculated with the Henderson–Hasselbalch equation, temperature-corrected pH, and temperature-corrected pCO_2_, with αCO_2_ and pK values calculated via species-specific equations for sea turtles (Stabenau and Heming 1993). The time lag (min) between collection of the blood sample (defined as the moment the sampling needle was removed from the turtle) and analysis in the i-STAT machine (defined as the moment the machine first displayed “Calibrating”) is reported in the Supplementary Material.

#### Complete blood counts

CBCs, including hematocrit, total WBC count, and WBC differential count, were determined within 18 h for pre-transport samples and control samples, and within 36 h for post-transport samples. Hematocrit was determined manually using standard capillary tube and centrifugation methods, whereas WBC was assessed using a hemocytometer and Phloxine B solution. Total leukocyte count was performed manually with a direct leukocyte counting method, and differential WBC counts were performed by a single board-certified veterinary clinical pathologist (D.D.) (for full methodological details see Hunt et al. 2016a). Of the CBC data, only WBC, H/L ratio, and hematocrit were analyzed statistically, since these three measures are known to increase during transport in Kemp’s ridleys and/or other vertebrates (Knowles and Warriss 2007; Hunt et al. 2016a, 2019); other CBC variables are reported in the Supplementary Material. Seventeen samples were not analyzed for all CBC variables due to shipping delays or hemolysis; these included four Kemp’s ridley samples (three post-18h-transport and one post-24h-transport) and 13 loggerhead samples (five pre-18h-transport, five pre-24h-transport, and three post-18h-transport).

#### Corticosterone assay

Unextracted plasma samples were assayed for corticosterone using a double-antibody ^125^I radioimmunoassay previously validated for Kemp’s ridley plasma (Hunt et al. 2012; catalog #07-120103, MP Biomedicals, Solon, OH, USA). As this assay has not before been validated for loggerhead plasma, we performed parallelism validations for loggerheads via assay of serial dilutions of pooled loggerhead plasma alongside a standard curve. Corticosterone assay followed the manufacturer’s protocol except that all samples, standards, and reagents were used at half-volume, and an additional low standard was added to the standard curve (created by mixing equal volumes of assay buffer and the manufacturer’s lowest standard), as in Hunt et al. (2016a, 2019). To keep samples within the range of the standard curve, turtle plasma samples were diluted 10-fold in assay buffer, with final results then multiplied by 10. Non-specific binding tubes and blanks were assayed in quadruplicate, and standards, low and high controls, and samples in duplicate. Any samples with a coefficient of variation >10% between duplicate tubes, or that fell outside 10–90% bound, were rediluted and reassayed accordingly. Intra- and inter-assay variations were both <10%. For further assay details, including antibody cross-reactivities and sensitivity, see Hunt et al. (2012, 2016a).

### Data analysis

Parallelism of the corticosterone assay for loggerhead plasma was assayed with an *F*-test comparing slope of the linear portions of the two curves (serially diluted loggerhead plasma pool vs. pure hormone standards). Descriptive statistics (median and mean±SEM or SD as appropriate) were used to summarize stress-associated and clinical health measures for both species, as well as actual transport durations, bleed time, body mass, and carapace measurements. Absolute data on corticosterone concentration, WBC, H/L ratio, and lactate concentration were log-transformed to adjust for non-normal distribution based on visual inspection of residual distributions and *Q*–*Q* plots. Lactate data were frequently below detectability limits (see the “Results” section) and for statistical analysis were assigned a nominal value of one-half the detectability limit. Variables were then divided into two multivariate datasets for analysis (as in Hunt et al. 2016a, 2019): (1) four “stress-associated measures” (“stress” data; corticosterone, glucose, WBC, and H/L ratio), i.e., variables consistently shown to increase significantly during ground transportation of sea turtles and (2) nine “clinical health measures” (“clinical” data; pH, pO_2_, pCO_2_, HCO_3_, sodium, potassium, iCa, lactate, and hematocrit), which are widely used to monitor clinical health. For stress-associated and clinical variables, we calculated the change in measured levels from the pre-transport sample (i.e., near baseline for an individual) to the post-transport sample for each turtle (paired before-after data). Resulting data represented the physiological response of turtles to experimental events, and better enabled exact comparisons by adjusting for any inherent differences in baseline physiology of turtle species. Physiological data were analyzed using multivariate linear mixed model (LMM) frameworks with normal probability distribution and identity link function. For each of the two datasets (i.e., stress-associated measures or clinical health measures), we conducted a series of LMM analyses that (1) determined the influence of control sampling (using pre- and post-event absolute data from control experiments for each species); (2) compared physiological responses of both species across the different event scenarios (control vs. transport and duration, using the full dataset, comparing percentage change from pre- to post-transport so as to enable cross-species comparisons); and (3) examined data for each species independently to better determine how different transport durations affect physiological responses in Kemp’s ridley and loggerhead turtles, when compared with responses from control events. A full factorial mixed model allowed both categorical fixed factors (i.e., species, event type [control or transport], pre- or post-event sampling, and transport duration) and random components (i.e., individual turtle and body mass [kg]) to be potentially fitted in analyses. Body mass was included as a covariate in all models, as differences in body condition and/or age could affect responses to environmental stressors (Moore and Jessop 2003; Jessop et al. 2004). *Post hoc* Bonferroni tests of pairwise multiple comparisons were then used to identify significant differences across transport durations for each species. Finally, to verify that the typical blood-sampling times of this study did not affect corticosterone in either species, we performed linear regression analyses of log-transformed corticosterone concentration against bleed time, examining the two species together and then separately. All data were also inspected by a veterinarian (C.I.) for any clinically relevant deviations from expected values for healthy individuals, when compared with data reported for juveniles of these species (Stabenau et al. 1991; Moon et al. 1997; Hoopes et al. 2000; Innis et al. 2007, 2009; Snoddy et al. 2009; Delgado et al. 2011; Fazio et al. 2012; Keller et al. 2012; Coleman et al. 2016; Hunt et al. 2016a, Stacy and Innis 2017). All statistical tests were conducted using SPSS^®^ statistical software (version 20.0 for Macintosh; SPSS Inc., Chicago, IL, USA) at a significance level of *P *<* *0.05.

## Results

### Assay parallelism

The corticosterone RIA exhibited good parallelism for loggerheads, with no significant difference in slope of the binding curves of serially diluted loggerhead plasma pool and corticosterone standards (*F*_1,8_* *=* *0.6595, *P *=* *0.4402).

### Transport durations

Actual transport event durations, defined as elapsed time between pre- and post-transport blood samples, sometimes differed slightly from planned durations due to unpredictable traffic and other logistical factors. Actual event durations for the four categories were as follows (control and transport events combined): <6 h events, 3.96 h ± 1.29 h (mean±standard deviation), range 2.3–5.9 h, *n *=* *8 events; ∼12 h events, 11.3 ± 1.2 h, range 10.0–12.9 h, *n *=* *6 events; ∼18 h events, 19.9 ± 1.0 h, range 18.1–20.7 h, *n *=* *5 events; ∼24 h events, 24.1 ± 0.9 h, range 23.1–25.9 h, *n *=* *8 events. For additional details, including event duration, date, year, and number of turtles in each event, see Supplementary Table S1.

### Effect of control sampling

Control sampling in the absence of transport had no overall significant effect in either species, indicating that factors such as handling, food restriction, and time-of-day do not greatly impact sea turtle physiology or clinical status (Kemp’s ridleys: stress data, *F*_4,61_=0.73, *P *=* *0.58; clinical data, *F*_9,52_=0.73, *P *=* *0.68; loggerheads: stress data, *F*_4,49_=0.59, *P *=* *0.67; clinical data, *F*_9,40_=1.22, *P *=* *0.31). When control-event variables were examined individually, *P *>* *0.05 for all measured variables except for iCa of loggerheads (*F*_1,48_=0.04, *P *=* *0.02) (Supplementary Tables S2–S5). These results help clarify that any differences in pre- versus post-transport measures would more likely be attributed to an effect of transport rather than an artifact of handling the turtles.

### Effect of transport

**Fig. 1:**
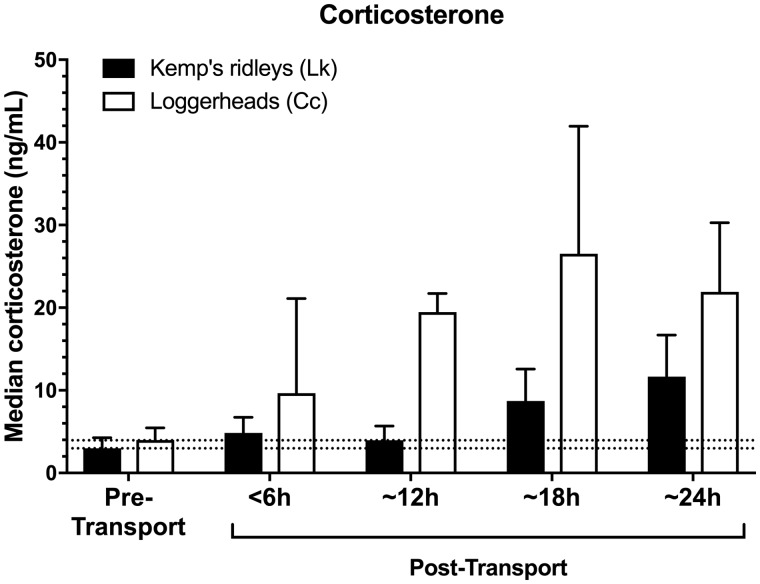
Post-transport corticosterone in Kemp’s ridley and loggerhead turtles when compared with pre-transport baselines. Dashed lines indicate pre-transport medians (i.e., bars on left), for visual comparison to post-transport concentrations in both species. Corticosterone was not normally distributed; bars indicate median±quartiles.

**Fig. 2:**
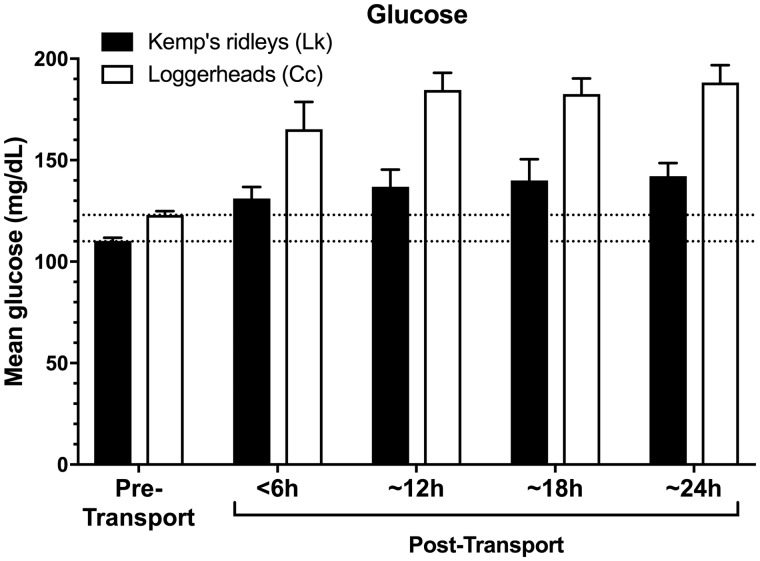
Post-transport glucose in Kemp’s ridley and loggerhead turtles when compared with pre-transport baselines. Dashed lines indicate pre-transport means (i.e., bars on left), for visual comparison to post-transport concentrations in both species. Bars indicate means±SEM.

**Fig. 3:**
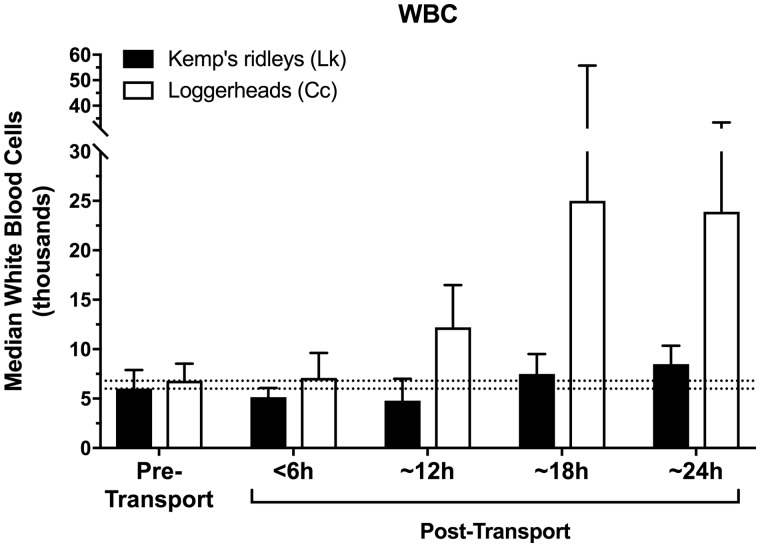
Post-transport WBC count in Kemp’s ridley and loggerhead turtles when compared with pre-transport baselines. Dashed lines indicate pre-transport medians (i.e., bars on left), for visual comparison to post-transport concentrations in both species. WBC data were not normally distributed; bars indicate median±quartiles.

**Fig. 4:**
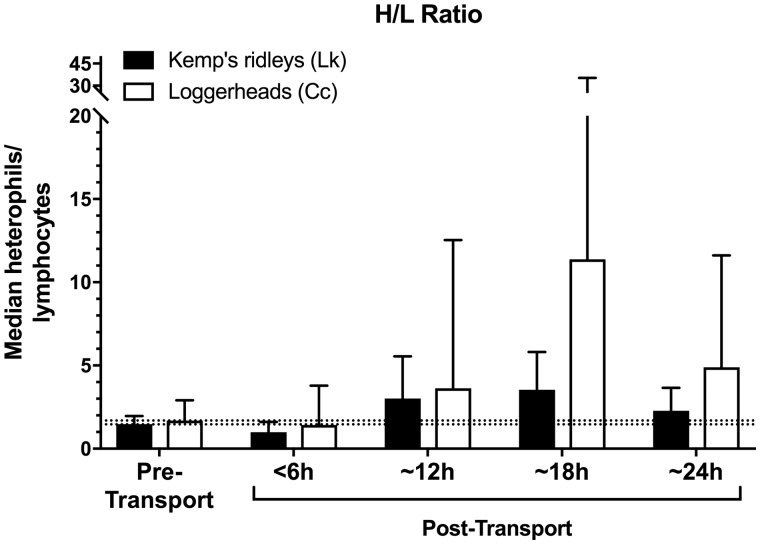
Post-transport heterophil/lymphocyte (H/L) ratio in Kemp’s ridley and loggerhead turtles when compared with pre-transport baselines. Dashed lines indicate pre-transport medians (i.e., bars on left), for visual comparison to post-transport concentrations in both species. H/L ratio was not normally distributed; bars indicate median±quartiles.

In contrast, transportation was found to markedly and significantly influence the physiological stress response of both sea turtle species (*F*_4,133_=30.12, *P *<* *0.001; Tables 1 and 2 and Figs. 1–4). When stress-related variables were examined individually, all four stress-related measures (corticosterone, glucose, WBC count, and H/L ratio) exhibited significant elevations above pre-transport levels in both species (all *P *<* *0.001; Tables 1 and 2 and Figs. 1–4). However, the effect of transport did not significantly influence the overall clinical health profile of the sea turtles (i.e., nine clinical health measures; *F*_9,113_=2.00, *P *=* *0.05; Tables 1 and 2). Loggerheads became mildly hyperglycemic during transports lasting 12 h or more (mean glucose ∼180 mg/dL); and developed a mild heterophilic leukocytosis during transports lasting 18 h or more (median WBC ∼20,000, mean heterophils ∼80%). After transport, all turtles remained in clinically healthy condition, appropriate for release, as judged by the attending veterinarians.

**Table 1:** Transport data from Kemp’s ridley turtles for four stress-associated variables and nine clinical health measures, before and after ground transport of four different durations

	Kemp’s ridley turtles—transport data							
	<6 h		∼12 h		∼18 h		∼24 h	
	Pre(*n *=* *8)	Post(*n *=* *8)	Pre(*n *=* *15)	Post(*n *=* *15)	Pre(*n *=* *8)	Post(*n *=* *8)	Pre(*n *=* *30)	Post(*n *=* *30)
Stress-associated measures								
Corticosterone* (ng/mL)	3.61(2.88–4.20)	4.86(3.02–6.74)	3.08(2.75–5.70)	3.96(2.79–5.69)	1.71(1.40–2.07)	8.71(6.43–12.57)	2.97(1.91–4.34)	11.67(8.06–16.68)
Glucose (mg/dL)	120.1±4.1	131.1±5.6	100.3±2.8	136.9±8.5	116.9±4.1	140.0±10.5	110.7±1.8	142.1±6.5
WBC count* (thousands)	6.60(5.25–9.23)	5.15(4.68–6.08)	5.50(4.20–6.40)	4.80(3.70–7.00)	6.15(4.95–7.50)	7.50(5.15–9.50)	5.85(4.28–8.35)	8.50 (*n* =29)(6.70–10.35)
H/L ratio*	1.74(0.77–1.90)	0.98(0.68–1.60)	1.72(1.20–2.63)	3.00(1.43–5.53)	1.24(1.05–1.43)	3.52(2.14–5.80)	1.31(1.00–1.93)	2.27 (*n*=29)(1.45–3.64)
Clinical health measures								
pH	7.57±0.02	7.59±0.01	7.49±0.01	7.49±0.01	7.55±0.02	7.54±0.04	7.53±0.01	7.54±0.01
pO_2_ (mm Hg)	74.3±3.3	75.0±3.1	62.8±2.2	69.5±2.9	86.2±2.7	85.4±5.7	71.7±1.6	67.4±1.7
pCO_2_ (mm Hg)	37.4±1.6	34.5±0.6	44.1±1.5	41.3±1.7	39.8±1.6	38.6±4.7	39.0±1.3	36.1±0.8
HCO_3_ (mmol/L)	40.6±1.0	39.9±0.8	37.5±1.0	36.4±0.8	41.0±1.0	38.6±1.0	37.3±0.7	37.1±0.7
Sodium (mmol/L)	149.8±1.5	149.0±2.3	150.1±0.6	149.2±0.7	148.9±1.0	152.3±1.0	150.9±0.5	151.5±0.6
Potassium (mmol/L)	3.33±0.07	3.25±0.08	3.88±0.11	3.65±0.16	3.24±0.15	3.11±0.12	3.49±0.09	3.25±0.12
Calcium (ionized; mmol/L)	0.88±0.04(*n* =6)	0.80±0.06(*n* =3)	0.92±0.03	0.87±0.03(*n* =14)	0.79±0.04(*n* =7)	0.78±0.04	0.84±0.01(*n* =29)	0.80±0.01(*n* =28)
Lactate* (mmol/L)	0.30(0.15–0.52)	0.15(0.15–0.46)	0.47(0.15–1.57)	0.15(0.15–0.89)	0.15(0.15–1.46)	0.15(0.15–4.12)	0.15(0.15–0.15)	0.15(0.15–0.15)
Hematocrit (%)	25.0±1.1	25.8±1.8	26.5±0.8	27.4±0.8	26.4±0.6	29.8±0.6	25.8±0.4	28.5±0.6

Number of turtles transported per duration is shown at top; exceptions with lower *n* (due to i-STAT cartridge failure, hemolysis, or shipping delays) are shown in the applicable cells. WBC, white blood cells; H/L, heterophils/lymphocytes. Mean±SEMs shown for normal data; median (25–75%) shown for non-normal data (marked with asterisk).

**Table 2:** Transport data from loggerhead turtles for four stress-associated variables and nine clinical health measures, before and after ground transport of four different durations

	Loggerhead turtles—transport data							
	<6 h		∼12 h		∼18 h		∼24 h	
	**Pre**(*n* = 8)	**Post**(*n* = 8)	**Pre**(*n* = 10)	**Post**(*n* = 10)	**Pre**(*n* = 8)	**Post**(*n* = 8)	**Pre**(*n* = 8)	**Post**(*n* = 8)
Stress-associated measures								
Corticosterone* (ng/mL)	3.58(2.64–6.20)	9.64(8.26–21.1)	4.93(3.53–5.84)	19.45(13.60–21.70)	3.69(3.12–4.59)	26.5(16.80–41.90)	3.71(3.02–5.54)	21.90 (*n* =6)(14.80–30.24)
Glucose (mg/dL)	122.9±4.7	165.3±13.4	121.6±4.1	184.6±8.4	123.0±2.7	182.6±7.7	124.9±4.1	188.3±8.6
WBC count* (thousands)	6.40(4.88–9.50)	7.10(4.93–9.63)	8.05(6.70–8.83)	12.20(9.18–16.50)	4.00 (*n* =3)(3.50–4.30)	25.00 (*n* =5)(22.30–55.75)	6.20 (*n* =3)(4.20–7.20)	23.90(12.18–33.45)
H/L ratio*	1.07(0.74–3.67)	1.43(0.51–3.79)	1.97(1.28–2.83)	3.63(2.29–12.5)	1.49(0.88–2.03)	11.38 (*n* =5)(7.68–35.25)	3.67 (*n* =7)(1.66–3.30)	4.89(3.99–11.61)
Clinical health measures								
pH (mm Hg)	7.57±0.01	7.58±0.02	7.55±0.01	7.55±0.01	7.57±0.01	7.54±0.01	7.59±0.01	7.59±0.01
pO_2_ (mm Hg)	64.6±3.1	63.1±3.0	64.7±2.2	59.6±5.4	68.8±2.3	56.7±2.9	64.2±3.7	59.7±2.2
pCO_2_ (mm Hg)	39.2±1.7	38.6±1.4	40.4±1.2	40.4±1.0	36.0±1.3	39.9±1.6	37.9±1.4	39.7±1.7
HCO_3_ (mmol/L)	42.7±1.3	43.2±1.5	41.2±0.9	39.7±0.9	40.4±1.5	41.0±1.0	44.3±1.3	46.4±1.0
Sodium (mmol/L)	155.1±0.7	154.6±0.63	154.1±0.7	154.7±0.6	153.0±0.6	153.5±0.3	153.9±0.9	156.1±0.8
Potassium (mmol/L)	3.33±0.07	3.51±0.15	3.45±0.07	3.51±0.21	3.28±0.10	3.46±0.18	3.46±0.11	3.39±0.10
Calcium (ionized; mmol/L)	0.92±0.02	0.86±0.03	0.92±0.02	0.88±0.01	0.85±0.01	0.85±0.03	0.84±0.02(*n* =7)	0.80±0.02
Lactate* (mmol/L)	0.15(0.15–0.49)	0.67(0.22–1.80)	0.15(0.15–0.38)	0.25(0.15–1.05)	0.15(0.15–0.15)	0.58(0.15–1.99)	0.31(0.15–0.71)	0.57(0.35–0.70)
Hematocrit (%)	31.9±1.3	33.9±1.6	32.0±1.2	32.3±0.9	31.3±1.2	34.4±1.2	31.5±0.7	35.3±1.2

Number of turtles transported per duration is shown at top; exceptions with lower *n* (due to i-STAT cartridge failure, hemolysis, or shipping delays) are shown in the applicable cells. WBC, white blood cells; H/L, heterophils/lymphocytes. Mean±SEMs shown for normal data; median (25–75%) shown for non-normal data (marked with asterisk).

### Effect of transport duration

Transport duration markedly and significantly affected the resulting stress responses of both species (*F*_12,352_=3.87, *P *<* *0.001; Tables 1 and 2 and Figs. 1–4), with both species generally showing more pronounced changes in the four stress-associated measures at longer durations of transport (*F*_12,352_=3.38, *P *=* *0.001; Figs. 1–4).

For loggerhead turtles, the longer transport durations of 18 and 24 h produced significantly greater elevations in WBC count (*F*_4,53_=17.78, *P *<* *0.001; Fig. 3) and H/L ratio (*F*_4,53_=3.58, *P *=* *0.01; Fig. 4) when compared with transports of 12 h or less. In contrast, loggerhead corticosterone (*F*_4,53_=16.08, *P *<* *0.001) and glucose concentrations (*F*_4,53_=32.57, *P *<* *0.001) were significantly elevated above control levels after even the shortest transport duration (<6 h), and both measures showed similar response levels across all transport durations, i.e., corticosterone and glucose appeared to respond maximally even at short transport durations, with no additional significant elevation at longer durations (Figs. 1 and 2).

For Kemp’s ridley turtles, WBC counts (*F*_4,85_=2.77, *P *=* *0.03; Fig. 3) and H/L ratios (*F*_4,85_=2.56, *P *=* *0.04; Fig. 4) were elevated after transports of 12 h or more duration, reaching maxima in the 24 h group. In contrast to loggerheads, corticosterone concentrations in Kemp’s ridleys were significantly elevated only after longer transport durations of 18 and 24 h (*F*_4,85_=7.99, *P *<* *0.001; Fig. 1), with maximal corticosterone in the 24 h group. Glucose concentrations of Kemp’s ridleys elevated significantly after 12 h and then showed no additional elevation at longer durations (*F*_4,85_=9.39, *P *<* *0.001; Fig. 2).

### Species differences

The two species had significantly different patterns of response to ground transport (stress: *F*_4,133_=11.39, *P *<* *0.001), with loggerheads exhibiting a more pronounced physiological stress response than Kemp’s ridleys in every one of the four stress-related measures (corticosterone, glucose, WBC, and H/L ratio) across all transport durations (all *P *<* *0.01; Tables 1 and 2 and Figs. 1–4). Specifically, transportation increased glucose levels in loggerheads by 47% compared with a 26% increase in Kemp’s ridleys; WBC count increased by 180% in loggerheads and 30% in Kemp’s ridleys; H/L ratio increased by 438% in loggerheads and 130% in Kemp’s ridleys; and corticosterone concentrations increased by 436% in loggerheads and 217% in Kemp’s ridleys. The highest corticosterone concentrations recorded in this study for any transport or control group, of either species, occurred in loggerheads after 18 h of transport (median of 26.5 ng/mL, range 10.18–113.33 ng/mL).

### Body size, timing parameters, and vital rates

As expected, loggerheads were significantly larger and heavier than Kemp’s ridleys (*P *<* *0.0001 for all comparisons). There was no overlap in body mass between individuals of the two species in this study. Loggerhead mean body mass was 31.7 ± 14.1 kg (±SD) (range 10.0–81.0 kg), mean SCL was 56.5 ± 9.0 cm (range 38.2–84.5 cm), and mean SCW was 48.3 ± 7.1 cm (range 34.2–66.3 cm). Kemp’s ridley mean body mass was 4.65 ± 1.30 kg (range 2.30–8.25 kg), mean SCL=30.7 ± 3.1 cm (range 24.0–38.1 cm), and mean SCW was 28.6 ± 3.2 cm (range 22.4–37.4 cm). Differences in body mass of turtles in this study did not significantly influence the observed physiological responses to transportation (all models *P *>* *0.05).

Bleed times were slightly, but significantly, longer for loggerheads than for Kemp’s ridleys (*U *=* *7948, *P *<* *0.0001), with a median bleed time for loggerheads of 2.35 min (range 0.65–18.23 min) when compared with median bleed time for Kemp’s ridleys of 1.87 min (range 0.63–10.25 min). However, there was no apparent relationship of bleed time to corticosterone in either species (Kemp’s ridleys, *F*_1,96_=0.2390, *P *=* *0.6260, *r*^2^ =0.002; loggerheads, *F*_1,63_=0.2097, *P *=* *0.6485, *r*^2^=0.003), i.e., bleed times were rapid enough in both species that corticosterone levels were unlikely to be affected by handling. In total, combining both species, bleed times were <3 min for 258 samples (78.9% of samples), <5 min for 305 samples (93.3%), and <10 min for 324 samples (99.1%), with only three samples exceeding bleed times of 10 min. The latter three samples include a Kemp’s ridley post-transport sample taken at 10.25 min after initial disturbance, a loggerhead post-transport sample taken at 12.95 min, and a loggerhead post-control sample taken at 18.23 min, the delays in all three cases due to difficulty obtaining blood. Even these three samples had corticosterone concentrations typical for the turtle’s species and experimental group. Given lack of evidence for any effect of bleed times in this study on corticosterone concentration, all corticosterone data were retained in the final dataset. Additional sample timing data, along with vital rates and detailed CBC data, are reported in the Supplementary Material for all transport and control events of both species.

#### Sample sizes and missing data

Final sample sizes of turtles are shown in Table 3. Over the course of this 7-year study, 328 turtle plasma samples were analyzed for 14 analytes, producing 4264 analyses in total. A few analyses of certain samples did not produce usable data, as follows: seventeen samples (5.2% of total samples) did not produce iCa data due to failure of the i-STAT cartridge for iCa only. Lactate was below the i-STAT detectability limit (<0.30 mmol/L) for the majority of samples, with 211 of the 328 samples (64.3%) having nondetectable lactate. A few samples could not be analyzed for WBC (*n *=* *17; 5.18% of total samples), H/L ratio (*n *=* *8; 2.44% of total samples), or corticosterone (*n *=* *2; 0.61% of total samples) due to hemolysis or unanticipated shipping delays. These sporadic cases of missing data were approximately equally distributed across species, event types, and event durations.

**Table 3:** Final sample sizes of individual turtles studied, summarized by event duration, event type (control/transport) and species

	Kemp’s ridleys		Loggerheads	
	Control	Transport	Control	Transport
<6 h duration	8	8	8	8
12 h duration	15	15	8	10
18 h duration	2	8	8	8
24 h duration	12	30	8	8

## Discussion

Kemp’s ridley and loggerhead turtles both show evidence of a consistent physiological stress response due to ground transport, i.e., significant elevations in four stress-associated variables (corticosterone, glucose, WBC, and H/L) after transports of at least ∼6 h (loggerheads) or 12 h (Kemp’s ridleys). Comparison to matched control data indicates that these changes are not attributable to effects of handling, repeated sampling, food deprivation, or time of day, but appear specifically due to the stimuli of transportation (i.e., out of water in an unfamiliar container, with associated noise and vibration) Such physiological responses to transport have been documented before in Kemp’s ridleys, but until now, no information on transport-related stress was available for other sea turtle species. Our data demonstrate that loggerheads also respond to transport, and, by extension, suggest that other sea turtle species may be affected by transport-related stress as well.

Species differences in physiological responses were evident, with loggerheads exhibiting more pronounced physiological changes at every transport duration than did the smaller Kemp’s ridleys. Even at the shortest transport durations tested (<6 h), some stress measures of loggerheads exceeded the 24 h maxima recorded in Kemp’s ridleys. Furthermore, loggerheads seemed to attain a maximum or near-maximum stress response at relatively shorter transport durations, with several stress-related variable measures elevating markedly and significantly at the shortest duration examined (<6 h) and plateauing thereafter. In contrast, Kemp’s ridleys showed milder changes that tended to increase incrementally with longer transport durations. It is possible that some of these differences may be due to species-specific variation in absolute concentrations of corticosterone, sensitivity to corticosterone, and, in general, clinical significance of these measures.

Overall, however, our loggerhead results, when compared with published data on stress physiology in these species (Gregory et al. 1996; Blanvillain et al. 2008), suggest that loggerheads experience a strong physiological stress response even after relatively short transport durations. The time course of corticosterone elevation during transport is unknown (i.e., post-transport corticosterone may not represent peak corticosterone during transport), but nonetheless, to our knowledge the post-transport corticosterone concentrations documented in this study are the highest corticosterone concentrations reported for loggerheads. Prior loggerhead studies report peak post-stressor corticosterone of ∼5.0 ng/mL for entanglement or ∼7.5 ng/mL for restraint (Gregory et al. 1996; Williard et al. 2015) with an individual maximum of 12.5 ng/mL (Williard et al. 2015). In our study, even the shortest transport duration, <6 h, produced a median post-transport corticosterone of 9.64 ng/mL, and all three longer durations produced median post-transport corticosterone near or above 20 ng/mL. At all four durations of transport, at least one loggerhead had corticosterone >20 ng/mL; in the two longest durations, several turtles had corticosterone >30 ng/mL. The highest corticosterone concentration observed in this study was from an individual loggerhead with corticosterone of 113 ng/mL after 18 h of transport, which appears to be the highest corticosterone concentration ever reported for this species. In addition, loggerheads became mildly hyperglycemic during transports lasting 12 h or more; and developed a mild heterophilic leukocytosis during transports lasting 18 h or more. Cumulatively, these data suggest that long-distance ground transport may represent a near-maximal stressor for loggerhead turtles. However, stress responses are presumed to be adaptive, and it is possible that the pronounced adrenal responses documented here for a single event (provided such events do not occur frequently) may not necessarily entail any negative consequences for individual loggerheads. Post-release monitoring was not possible in this study (due in part to the fact that attachment of transmitters can affect corticosterone; K. E. Hunt, unpublished data). In the future, long-term data on post-release movement and survival would help clarify whether loggerheads remain in good health subsequent to release after prolonged transports.

For Kemp’s ridleys, only the more prolonged transport durations of at least 12–18 h caused significant increases in glucose, corticosterone, and H/L ratio, while WBC counts did not significantly elevate until 24 h. Comparisons to published literature on this species suggest that transportation did not induce a maximum physiological stress response in Kemp’s ridleys, even after 24 h of transport (Ortiz et al. 2000; Gregory and Schmid 2001; Hunt et al. 2012). Following terminology of Hunt et al. (2012, 2016a, 2016b, 2019), we suggest that transports of 24 h or less, using the protocols described here, represent a moderate but not a maximal stressor for Kemp’s ridleys.

Importantly, despite the significant effect of transport on stress-related measures, other clinical measures of health (heart rate, respiratory rate, acid–base status, and electrolytes) remained within acceptable limits for both species and the turtles completed transport in good condition. Nonetheless, in the absence of data to the contrary, we suggest erring on the side of caution when transporting loggerheads (and perhaps other larger-bodied sea turtles), i.e., minimizing transport duration when possible.

## Conclusions

Recent analyses have suggested a potential causal relationship between ongoing increases in sea surface temperature and the simultaneous increases in turtle stranding numbers on Cape Cod (Griffin et al. 2019). The number of sea turtles stranding annually on Cape Cod is therefore predicted to continue to increase in the coming decades (Griffin et al. 2019). Given the continued vulnerable status of sea turtle species in US waters (IUCN 2019; USFWS 2019), rehabilitation efforts are likely to continue, and thus turtle transport events are likely to continue as well. Inevitably, many of these transports will require transportation to a quite distant location; 18 h and occasional 24 h transports to Georgia and Florida will probably always be necessary. While our data suggest that such transports will stress sea turtles in a physiological sense (i.e., activation of the HPA axis), our study shows that turtles of both species nevertheless can remain in good clinical condition despite prolonged transport times. Health measures for the majority of individuals in this study remained within clinically acceptable limits, and, anecdotally, all turtles crawled vigorously and swam well upon release to sea. Thus, the transport protocols utilized in this study appear clinically safe for transports of up to 24 h in duration.

However, the documented negative long-term effects of elevated corticosterone in other species warrant continued attention to this issue. We suggest that in the absence of data on potential long-term effects of corticosterone in chelonians, it would be advisable to minimize transport stress where possible. In our experience, transport duration, and presumably associated stress, can be considerably minimized with attention to the logistical details of a sea turtle transport event. Feasible and effective changes to reduce transport time from the NEAq clinic have included: scheduling additional staff to reduce pre-transport preparation time, scheduling transports to reduce chances of encountering heavy traffic at major cities en route, eliminating meal stops, shortening refueling stops, scheduling sufficient additional staff or vehicles to enable driving throughout the night, and encouraging efficient use of time for any necessary media, public-relations, and research activities during subsequent beach releases. Our pre-transport preparation protocols (administration of subcutaneous fluids, usage of lubricant) may also be a factor in promoting good clinical condition even during longer transports. The transport environment, too, is likely important; our routine protocols include placing turtles in padded, dim, individual containers; minimizing handling, conversational noise, vehicle noise, and unnecessary acceleration/deceleration; usage of dim lighting in turtle cargo areas; and continuous monitoring and adjustment of cargo-area temperature. While our study did not specifically test the effect of these factors, we consider it likely that they help minimize stress. We note, also, that it is possible that same-day transports might have differing physiological effects than overnight transports (the present study was not designed to separate effects of an overnight phase from transport duration). Overall, we recommend that clinicians and veterinarians continue to study and adjust sea turtle transport protocols so as to achieve maximum health and minimal physiological stress for a given transport duration, with the ultimate goal of releasing sea turtles to sea in ideal or near-ideal condition. Future studies should seek to characterize short-term and long-term post-release behavior and outcomes after various transport events, to enable further refinements in transportation protocols.

## Supplementary Material

obaa012_Supplementary_Data
