# Supplementary material for: Effects of Ground Transport in Kemp’s Ridley (Lepidochelys kempii) and Loggerhead (Caretta caretta) Turtles
Source: Integr Org Biol. 2020 May 19;2(1):obaa012. doi: 10.1093/iob/obaa012 (PMC7671109; doi:10.1093/iob/obaa012)
Supplement: obaa012_Supplementary_Data [file obaa012_supplementary_data.zip › Table S1.docx]

**Table S1.** Dates, timing, durations and sample sizes of all control events and transport events. Duration bin indicates planned duration of the event. Actual duration = mean of elapsed time from first blood sample to second blood sample, calculated for each turtle individually and averaged for the entire event. (Actual duration may differ slightly from duration bin due to traffic, weather, and other logistical factors.) Target sample size was *n* = 8 turtles per species, event type, and event duration; higher *n*'s for some Kemp's ridley events are due to pilot studies and/or concurrent research on other aspects of sea turtle transportation.

| **Event Type** | **Duration Bin (h)** | **Start Date** | **Actual Duration (h) (mean ± st dev)** | **Kemp's Ridleys (n)** | **Loggerheads (n)** |
| --- | --- | --- | --- | --- | --- |
| Control | <6 | 03-Feb-2014 | 5.87 ± 0.15 | 0 | 3 |
| Control | <6 | 21-May-2014 | 4.14 ± 0.05 | 0 | 4 |
| Control | <6 | 15-Mar-2017 | 4.67 ± 0.04 | 8 | 1 |
| Control | ~12 | 23-May-2012 | 12.32 ± 0.29 | 15 | 2 |
| Control | ~12 | 16-Jun-2014 | 10.18 ± 0.05 | 0 | 4 |
| Control | ~12 | 03-May-2017 | 11.82 ± 0.05 | 0 | 2 |
| Control | ~18 | 22-Feb-2017 | 20.38 ± 0.03 | 2 | 4 |
| Control | ~18 | 11-Apr-2017 | 18.15 ± 0.05 | 0 | 4 |
| Control | ~24 | 20-Mar-2013 | 23.96 ± 0.01 | 12 | 2 |
| Control | ~24 | 14-Apr-2014 | 23.11 ± 0.28 | 0 | 2 |
| Control | ~24 | 21-Jun-2017 | 23.54 ± 0.05 | 0 | 3 |
| Control | ~24 | 05-Feb-2018 | 24.02 ± 0.00 | 0 | 1 |
| Transport | <6 | 07-May-2014 | 4.23 ± 0.13 | 0 | 4 |
| Transport | <6 | 06-Jun-2017 | 5.09 ± 0.02 | 4 | 0 |
| Transport | <6 | 15-Aug-2017 | 2.85 ± 0.04 | 0 | 2 |
| Transport | <6 | 23-Aug-2017 | 2.54 ± 0.02 | 0 | 2 |
| Transport | <6 | 14-Aug-2018 | 2.28 ± 0.06 | 4 | 0 |
| Transport | ~12 | 10-Jun-2012 | 12.91 ± 0.14 | 15 | 2 |
| Transport | ~12 | 22-Jun-2014 | 10.86 ± 0.06 | 0 | 4 |
| Transport | ~12 | 22-Jun-2016 | 9.96 ± 0.04 | 0 | 4 |
| Transport | ~18 | 05-Mar-2017 | 20.50 ± 0.02 | 3 | 3 |
| Transport | ~18 | 22-May-2017 | 20.72 ± 0.03 | 5 | 0 |
| Transport | ~18 | 22-Apr-2018 | 19.85 ± 0.04 | 0 | 5 |
| Transport | ~24 | 06-Apr-2013 | 25.89 ± 0.02 | 12 | 1 |
| Transport | ~24 | 21-Apr-2014 | 24.89 ± 0.04 | 0 | 2 |
| Transport | ~24 | 15-May-2015 | 23.99 ± 0.00 | 18 | 0 |
| Transport | ~24 | 22-Apr-2018 | 23.65 ± 0.07 | 0 | 5 |
