# Supplementary material for: Effects of Ground Transport in Kemp’s Ridley (Lepidochelys kempii) and Loggerhead (Caretta caretta) Turtles
Source: Integr Org Biol. 2020 May 19;2(1):obaa012. doi: 10.1093/iob/obaa012 (PMC7671109; doi:10.1093/iob/obaa012)
Supplement: obaa012_Supplementary_Data [file obaa012_supplementary_data.zip › Table S2.docx]

**Table S2.** Kemp's ridley control-event data for stress-associated and clinical health measures. Number of turtles studied per duration is shown at top. WBC = white blood cells; H/L = heterophils/lymphocytes. Mean ± SEMs shown for normal data; medians and interquartiles shown for non-normal data (indicated with asterisk).

|  | **KEMP'S RIDLEY TURTLES - CONTROL DATA** | | | | | | | |
| --- | --- | --- | --- | --- | --- | --- | --- | --- |
|  | **<6 h** | | **~12 h** | | **~18 h** | | **~24 h** | |
|  | **Pre**  (*n*=8) | **Post**  (*n*=8) | **Pre**  *(n*=15) | **Post**  (*n*=15) | **Pre**  (*n*=2) | **Post**  (*n*=2) | **Pre**  (*n*=12) | **Post**  (*n*=12) |
| ***1. Stress-associated measures*** | | | | | | | | |
| **Corticosterone***  (ng/mL) | 3.44  (2.45-4.43) | 2.76  (2.48-4.29) | 2.41  (1.94-2.87) | 2.85  (2.12-4.54) | 2.38  (1.05-3.71) | 2.10  (1.58-2.62) | 1.70  (1.41-2.05) | 3.26  (1.87-4.58) |
| **Glucose**  (mg/dL) | 125.5 ± 2.5 | 120.0 ± 5.9 | 110.2 ± 2.0 | 114.6 ± 3.2 | 121.0 ± 17.0 | 126.0 ± 17.0 | 104.8 ± 2.6 | 111.8 ± 2.5 |
| **WBC Count***  (thousands) | 4.20  (4.03-5.08) | 4.40  (3.83-4.58) | 4.40  (3.90-5.10) | 4.60  (3.80-6.10) | 7.15  (5.00-9.30) | 4.90  (3.70-6.10) | 5.85  (4.04-7.53) | 6.55  (5.53-9.15) |
| **H/L Ratio*** | 1.27  (1.18-1.63) | 1.52  (1.20-1.67) | 2.16  (1.49-2.63) | 2.54  (1.57-3.90) | 0.90  (0.90-0.90) | 0.98  (0.90-1.06) | 1.07  (0.61-1.78) | 1.17  (0.76-1.55) |
| ***2. Clinical health measures*** | | | | | | | | |
| **pH** | 7.53 ± 0.03 | 7.58 ± 0.02 | 7.54 ± 0.01 | 7.51 ± 0.01 | 7.56 ± 0.01 | 7.56 ± 0.01 | 7.53 ± 0.02 | 7.52 ± 0.02 |
| **pO_2_**  (mm Hg) | 77.8 ± 6.1 | 83.3 ± 6.0 | 67.9 ± 2.2 | 62.3 ± 2.0 | 88.0 ± 8.5 | 87.6 ± 3.8 | 67.2 ± 2.4 | 72.3 ± 3.6 |
| **pCO_2_**  (mm Hg) | 38.3 ± 2.7 | 34.6 ± 1.2 | 39.8 ± 0.8 | 41.0 ± 1.2 | 41.3 ± 2.3 | 35.5 ± 2.8 | 38.4 ± 2.2 | 37.4 ± 2.3 |
| **HCO_3_**  (mmol/L) | 37.7 ± 0.8 | 38.9 ± 1.0 | 38.8 ± 1.0 | 37.8 ± 1.0 | 42.9 ± 1.9 | 37.2 ± 1.7 | 37.2 ± 1.1 | 35.4 ± 2.3 |
| **Sodium**  (mmol/L) | 150.0 ± 1.6 | 149.9 ± 1.2 | 148.9 ± 0.8 | 150.1 ± 0.6 | 150.0 ± 0.0 | 145.0 ± 5.0 | 152.3 ± 0.6 | 152.9 ± 0.4 |
| **Potassium**  (mmol/L) | 3.79 ± 0.21 | 3.45 ± 0.20 | 3.42 ± 0.04 | 3.79 ± 0.13 | 3.25 ± 0.15 | 3.05 ± 0.05 | 3.69 ± 0.22 | 3.75 ± 0.23 |
| **Calcium**  (ionized; mmol/L) | 0.81 ± 0.03 | 0.82 ± 0.04 | 0.88 ± 0.02 | 0.85 ± 0.03 | 0.78 ± 0.07 | 0.78 ± 0.05 | 0.86 ± 0.02 | 0.82 ± 0.02 |
| **Lactate***  (mmol/L) | 1.65  (0.15-2.88) | 0.15  (0.15-1.43) | 0.15  (0.15-0.15) | 0.51  (0.15-2.06) | 0.38  (0.15-0.60) | 0.15  (0.15-0.15) | 0.15  (0.15-1.64) | 0.15  (0.15-2.21) |
| **Hematocrit** (%) | 29.3 ± 0.7 | 28.7 ± 0.9 | 29.3 ± 0.6 | 28.5 ± 0.6 | 30.0 ± 3.0 | 29.5 ± 2.5 | 28.5 ± 0.7 | 27.4 ± 0.9 |
