# Supplementary material for: Effects of Ground Transport in Kemp’s Ridley (Lepidochelys kempii) and Loggerhead (Caretta caretta) Turtles
Source: Integr Org Biol. 2020 May 19;2(1):obaa012. doi: 10.1093/iob/obaa012 (PMC7671109; doi:10.1093/iob/obaa012)
Supplement: obaa012_Supplementary_Data [file obaa012_supplementary_data.zip › Table S3.docx]

**Table S3.** Kemp's ridley control-event data for vital rates, hematology and timing. Number of turtles studied per duration is shown at top. Mean ± SEMs (vital rates, hematologic data) or mean ± standard deviations (timing data) are shown in each cell.

|  | **KEMP'S RIDLEY TURTLES - CONTROL DATA** | | | | | | | |
| --- | --- | --- | --- | --- | --- | --- | --- | --- |
|  | **<6 h** | | **~12 h** | | **~18 h** | | **~24 h** | |
|  | **Pre**  (*n*=8) | **Post**  (*n*=8) | **Pre**  *(n*=15) | **Post**  (*n*=15) | **Pre**  (*n*=2) | **Post**  (*n*=2) | **Pre**  (*n*=12) | **Post**  (*n*=12) |
| ***1. Vital rates*** | | | | | | | | |
| **Cloacal Temp.** (°C) | 24.2 ± 0.1 | 23.9 ± 0.1 | 26.9 ± 0.1 | 26.4 ± 0.1 | 25.2 ± 0.0 | 25.3 ± 0.1 | 24.5 ± 0.1 | 24.4 ± 0.1 |
| **Heart Rate**  (bpm) | 45.3 ± 1.9 | 42.6 ± 0.7 | 52.8 ± 1.2 | 51.6 ± 1.1 | 48.5 ± 0.5 | 46.0 ± 2.0 | 48.8 ± 0.9 | 46.0 ± 1.0 |
| **Respiration**  (per min) | 2.8 ± 0.3 | 3.0 ± 0.4 | 4.9 ± 0.6 | 3.9 ± 0.3 | 5.0 ± 2.0 | 4.0 ± 1.0 | 4.8 ± 0.7 | 3.8 ± 0.5 |
| ***2. Hematologic data*** | | | | | | | | |
| **Heterophils (%)** | 54.4 ± 2.1 | 58.6 ± 3.2 | 64.9 ± 2.0 | 68.9 ± 2.5 | 45.0 ± 2.0 | 48.5 ± 1.5 | 48.0 ± 4.0 | 50.0 ± 3.0 |
| **Lymphocytes (%)** | 40.4 ± 2.3 | 38.4 ± 2.9 | 32.5 ± 1.7 | 29.0 ± 2.4 | 50.0 ± 2.0 | 49.5 ± 2.5 | 47.8 ± 4.0 | 46.7 ± 2.7 |
| **Monocytes (%)** | 5.0 ± 1.2 | 2.9 ± 0.9 | 1.9 ± 0.3 | 1.3 ± 0.2 | 5.0 ± 4.0 | 2.0 ± 1.0 | 2.0 ± 0.3 | 1.7 ± 0.5 |
| **Eosinophils (%)** | 0.3 ± 0.2 | 0.1 ± 0.1 | 0.7 ± 0.2 | 0.7 ± 0.2 | 0.0 ± 0.0 | 0.0 ± 0.0 | 2.1 ± 0.5 | 1.7 ± 0.5 |
| **Heterophils** (cells/uL) | 2453 ± 210 | 2493 ± 175 | 2877 ± 203 | 3789 ± 570 | 3175 ± 825 | 2359 ± 509 | 2686 ± 231 | 3821 ± 531 |
| **Lymphocytes** (cells/uL) | 1815 ± 169 | 1621 ± 130 | 1412 ± 88 | 1466 ± 152 | 3532 ± 932 | 2456 ± 717 | 2966 ± 439 | 3463 ± 359 |
| **Monocytes** (cells/uL) | 220 ± 53 | 118 ± 33 | 85 ± 16 | 63 ± 11 | 444 ± 394 | 86 ± 25 | 104 ± 13 | 155 ± 68 |
| **Eosinophils** (cells/uL) | 12 ± 8 | 6 ± 6 | 33 ± 8 | 26 ± 8 | 0 ± 0 | 0 ± 0 | 122 ± 38 | 111 ± 37 |
| ***3. Timing data*** | | | | | | | | |
| **Bleed time** (min) | 2.20 ± 0.32 | 2.58 ± 0.55 | 1.94 ± 0.08 | 3.05 ± 1.37 | 2.57 ± 0.24 | 2.02 ± 0.21 | 2.66 ± 1.91 | 2.42 ± 1.23 |
| **Handling time**  (min) | 8.25 ± 1.00 | 7.71 ± 1.13 | 11.98 ± 0.72 | 9.63 ± 2.05 | 7.68 ± 0.22 | 7.64 ± 0.32 | 7.63 ± 1.99 | 6.83 ± 2.09 |
| **CG4 lag time** (min) | 1.35 ± 0.50 | 1.35 ± 0.64 | 2.54 ± 0.50 | 1.52 ± 0.55 | 0.98 ± 0.07 | 3.66 ± 3.74 | 2.33 ± 1.32 | 1.59 ± 0.53 |
| **CG8 lag time**  (min) | 5.68 ± 1.28 | 5.49 ± 1.24 | 7.01 ± 0.81 | 5.24 ± 0.54 | 5.02 ± 0.01 | 3.83 ± 1.33 | 6.45 ± 1.46 | 5.44 ± 0.58 |
