# Supplementary material for: Effects of Ground Transport in Kemp’s Ridley (Lepidochelys kempii) and Loggerhead (Caretta caretta) Turtles
Source: Integr Org Biol. 2020 May 19;2(1):obaa012. doi: 10.1093/iob/obaa012 (PMC7671109; doi:10.1093/iob/obaa012)
Supplement: obaa012_Supplementary_Data [file obaa012_supplementary_data.zip › Table S4.docx]

**Table S4.** Loggerhead control-event data for stress-associated and clinical health measures. Number of turtles studied per duration is shown at top. WBC = white blood cells; H/L = heterophils/lymphocytes. Mean ± SEMs shown for normal data; medians and interquartiles shown for non-normal data (indicated with asterisk).

|  | **LOGGERHEAD TURTLES - CONTROL DATA** | | | | | | | |
| --- | --- | --- | --- | --- | --- | --- | --- | --- |
|  | **<6 h** | | **~12 h** | | **~18 h** | | **~24 h** | |
|  | **Pre**  (*n*=8) | **Post**  (*n*=8) | **Pre**  *(n*=8) | **Post**  (*n*=8) | **Pre**  (*n*=8) | **Post**  (*n*=8) | **Pre**  (*n*=8) | **Post**  (*n*=8) |
| ***1. Stress-associated measures*** | | | | | | | | |
| **Corticosterone***  (ng/mL) | 4.32  (3.49-9.34) | 3.26  (2.97-4.59) | 3.72  (3.11-4.58) | 3.71  (1.85-4.65) | 2.36  (2.23-2.48) | 3.75  (2.40-6.82) | 5.54  (4.60-6.62) | 4.80  (3.19-6.15) |
| **Glucose**  (mg/dL) | 118.0 ± 4.5 | 126.6 ± 3.8 | 121.0 ± 4.3 | 131.3 ± 6.9 | 126.3 ± 5.2 | 127.0 ± 3.4 | 119.9 ± 5.7 | 120.1 ± 4.3 |
| **WBC Count***  (thousands) | 6.50  (4.83-9.00) | 5.80  (4.80-8.60) | 7.65  (6.13-9.15) | 7.45  (6.70-10.73) | 5.05  (4.28-6.13) | 4.95  (3.90-5.15) | 6.50  (5.83-7.45) | 6.05  (4.70-7.90) |
| **H/L Ratio*** | 1.80  (0.88-2.85) | 2.24  (1.03-3.55) | 1.67  (1.40-2.66) | 1.94  (1.04-2.86) | 0.84  (0.75-1.69) | 1.03  (0.79-1.33) | 1.17  (0.99-2.01) | 0.93  (0.55-1.61) |
| ***2. Clinical health measures*** | | | | | | | | |
| **pH** | 7.51 ± 0.01 | 7.53 ± 0.02 | 7.54 ± 0.01 | 7.52 ± 0.01 | 7.54 ± 0.02 | 7.53 ± 0.01 | 7.57 ± 0.02 | 7.57 ± 0.02 |
| **pO_2_**  (mm Hg) | 59.6 ± 2.6 | 51.6 ± 3.2 | 55.4 ± 5.5 | 60.8 ± 3.0 | 70.7 ± 5.4 | 68.7 ± 1.9 | 69.1 ± 4.6 | 69.1 ± 2.9 |
| **pCO_2_**  (mm Hg) | 44.3 ± 2.4 | 43.1 ± 2.3 | 41.4 ± 1.6 | 44.5 ± 1.8 | 39.7 ± 2.1 | 42.2 ± 1.6 | 42.8 ± 3.3 | 42.1 ± 2.2 |
| **HCO_3_**  (mmol/L) | 40.7 ± 1.5 | 42.0 ± 1.2 | 41.1 ± 1.2 | 41.8 ± 1.7 | 39.3 ± 1.6 | 39.9 ± 1.5 | 45.8 ± 2.1 | 44.7 ± 1.6 |
| **Sodium**  (mmol/L) | 154.9 ± 1.0 | 156.1 ± 0.6 | 154.6 ± 1.2 | 154.1 ± 0.8 | 154.1 ± 1.1 | 155.1 ± 0.8 | 153.8 ± 0.9 | 155.1 ± 0.6 |
| **Potassium**  (mmol/L) | 3.44 ± 0.07 | 3.55 ± 0.16 | 3.56 ± 0.11 | 3.39 ± 0.11 | 3.49 ± 0.11 | 3.20 ± 0.10 | 3.30 ± 0.09 | 3.25 ± 0.11 |
| **Calcium**  (ionized; mmol/L) | 0.92 ± 0.04 | 0.87 ± 0.03 | 0.93 ± 0.03 | 0.90 ± 0.03 | 0.86 ± 0.02 | 0.85 ± 0.01 | 0.94 ± 0.05 | 0.82 ± 0.04 |
| **Lactate***  (mmol/L) | 0.30  (0.29-0.37) | 0.36  (0.29-0.71) | 0.30  (0.29-0.89) | 0.29  (0.29-0.30) | 0.30  (0.30-0.77) | 0.30  (0.30-0.43) | 0.30  (0.29-0.47) | 0.30  (0.29-0.31) |
| **Hematocrit** (%) | 34.4 ± 1.5 | 34.3 ± 1.2 | 36.1 ± 1.8 | 35.9 ± 1.2 | 33.7 ± 1.1 | 34.0 ± 1.0 | 35.2 ± 0.7 | 35.8 ± 1.4 |
