# Supplementary material for: Effects of Ground Transport in Kemp’s Ridley (Lepidochelys kempii) and Loggerhead (Caretta caretta) Turtles
Source: Integr Org Biol. 2020 May 19;2(1):obaa012. doi: 10.1093/iob/obaa012 (PMC7671109; doi:10.1093/iob/obaa012)
Supplement: obaa012_Supplementary_Data [file obaa012_supplementary_data.zip › Table S5.docx]

**Table S5.** Loggerhead control-event data for vital rates, hematology and timing. Number of turtles studied per duration is shown at top. Mean ± SEMs (vital rates, hematologic data) or mean ± standard deviations (timing data) are shown in each cell.

|  | **LOGGERHEAD TURTLES - CONTROL DATA** | | | | | | | |
| --- | --- | --- | --- | --- | --- | --- | --- | --- |
|  | **<6 h** | | **~12 h** | | **~18 h** | | **~24 h** | |
|  | **Pre**  (*n*=8) | **Post**  (*n*=8) | **Pre**  *(n*=8) | **Post**  (*n*=8) | **Pre**  (*n*=8) | **Post**  (*n*=8) | **Pre**  (*n*=8) | **Post**  (*n*=8) |
| ***1. Vital rates*** | | | | | | | | |
| **Cloacal Temp.** (°C) | 25.5 ± 0.1 | 25.4 ± 0.1 | 25.7 ± 0.4 | 26.0 ± 0.3 | 25.4 ± 0.2 | 25.3 ± 2.0 | 25.1 ± 0.1 | 24.8 ± 0.2 |
| **Heart Rate**  (bpm) | 30.6 ± 3.2 | 34.0 ± 2.6 | 37.0 ± 2.7 | 41.0 ± 2.7 | 36.1 ± 2.3 | 36.8 ± 2.1 | 35.3 ± 2.3 | 32.6 ± 3.0 |
| **Respiration**  (per min) | 2.5 ± 0.4 | 3.0 ± 0.4 | 3.0 ± 0.3 | 3.6 ± 0.2 | 2.4 ± 0.6 | 2.1 ± 0.3 | 2.9 ± 0.6 | 2.4 ± 0.6 |
| ***2. Hematologic data*** | | | | | | | | |
| **Heterophils (%)** | 54.4 ± 5.1 | 56.9 ± 4.7 | 58.0 ± 3.0 | 58.6 ± 5.3 | 46.5 ± 3.9 | 47.3 ± 3.5 | 51.9 ± 5.0 | 44.4 ± 5.1 |
| **Lymphocytes (%)** | 38.3 ± 5.9 | 32.8 ± 5.5 | 33.1 ± 3.5 | 32.8 ± 4.2 | 45.4 ± 3.5 | 44.4 ± 3.2 | 38.6 ± 4.3 | 45.3 ± 4.4 |
| **Monocytes (%)** | 3.3 ± 0.5 | 4.8 ± 0.6 | 2.4 ± 0.7 | 2.1 ± 0.5 | 4.4 ± 1.1 | 2.5 ± 0.7 | 3.5 ± 0.8 | 2.4 ± 0.3 |
| **Eosinophils (%)** | 4.1 ± 1.1 | 5.6 ± 2.3 | 6.5 ± 1.8 | 6.5 ± 1.6 | 3.8 ± 0.9 | 5.9 ± 1.7 | 6.0 ± 1.8 | 8.0 ± 4.0 |
| **Heterophils** (cells/uL) | 3988 ± 650 | 3968 ± 832 | 4807 ± 849 | 4756 ± 596 | 2484 ± 359 | 2389 ± 429 | 3536 ± 271 | 2878 ± 263 |
| **Lymphocytes** (cells/uL) | 2783 ± 633 | 2274 ± 523 | 2589 ± 299 | 2696 ± 438 | 2304 ± 159 | 2082 ± 135 | 3159 ± 884 | 3379 ± 736 |
| **Monocytes** (cells/uL) | 252 ± 59 | 340 ± 73 | 233 ± 91 | 187 ± 61 | 229 ± 59 | 124 ± 35 | 238 ± 51 | 169 ± 32 |
| **Eosinophils** (cells/uL) | 303 ± 78 | 518 ± 308 | 583 ± 183 | 550 ± 163 | 183 ± 39 | 281 ± 87 | 580 ± 306 | 1013 ± 758 |
| ***3. Timing data*** | | | | | | | | |
| **Bleed time** (min) | 2.36 ± 1.06 | 2.95 ± 2.23 | 2.95 ± 1.42 | 2.09 ± 0.82  (n=7) | 3.18 ± 1.16 | 3.10 ± 2.19 | 3.87 ± 2.17 | 3.69 ± 2.29 |
| **Handling time**  (min) | 8.79 ± 1.14 | 8.44 ± 1.38  (*n*=7) | 10.87 ± 3.11 | 7.66 ± 1.89  (n=7) | 10.16 ± 1.87 | 7.88 ± 2.47 | 10.40 ± 3.22 | 10.15 ± 4.50  (n=7) |
| **CG4 lag time** (min) | 1.89 ± 0.75 | 1.74 ± 1.18 | 1.73 ± 0.67 | 1.39 ± 0.32  (n=7) | 2.06 ± 2.20 | 2.18 ± 1.94 | 1.71 ± 0.25  (n=7) | 1.90 ± 0.97  (n=7) |
| **CG8 lag time**  (min) | 6.31 ± 1.08 | 5.51 ± 1.21 | 5.80 ± 0.82 | 5.39 ± 0.55  (n=7) | 5.94 ± 1.49 | 6.19 ± 2.11 | 5.82 ± 0.64 | 6.92 ± 2.14 |
