# Supplementary material for: Effects of Ground Transport in Kemp’s Ridley (Lepidochelys kempii) and Loggerhead (Caretta caretta) Turtles
Source: Integr Org Biol. 2020 May 19;2(1):obaa012. doi: 10.1093/iob/obaa012 (PMC7671109; doi:10.1093/iob/obaa012)
Supplement: obaa012_Supplementary_Data [file obaa012_supplementary_data.zip › Table S6.docx]

**Table S6.** Kemp's ridley transport-event data for vital rates, hematology and timing. Number of turtles studied per duration is shown at top; exceptions with lower *n* (e.g., due to i-STAT cartridge failure, hemolysis, or shipping delays) are shown in the applicable cells. Mean ± SEMs (vital rates, hematologic data) or mean ± standard deviations (timing data) are shown in each cell.

|  | **KEMP'S RIDLEY TURTLES - TRANSPORT DATA** | | | | | | | |
| --- | --- | --- | --- | --- | --- | --- | --- | --- |
|  | **<6 h** | | **~12 h** | | **~18 h** | | **~24 h** | |
|  | **Pre**  (*n*=8) | **Post**  (*n*=8) | **Pre**  *(n*=15) | **Post**  (*n*=15) | **Pre**  (*n*=8) | **Post**  (*n*=8) | **Pre**  (*n*=30) | **Post**  (*n*=30) |
| ***1. Vital rates*** | | | | | | | | |
| **Cloacal Temp.** (°C) | 24.4 ± 0.1 | 23.3 ± 0.5 | 27.8 ± 0.1 | 25.6 ± 0.3 | 23.9 ± 0.1 | 23.1 ± 0.7 | 25.1 ± 0.1 | 23.6 ± 0.2 |
| **Heart Rate**  (bpm) | 41.3 ± 3.0 | 35.9 ± 2.6 | 55.0 ± 1.7 | 48.0 ± 1.4 | 37.6 ± 1.2 | 37.5 ± 2.1 | 47.4 ± 0.7 | 39.5 ± 1.0 |
| **Respiration**  (per min) | 2.9 ± 0.6 | 3.4 ± 0.7 | 3.0 ± 0.4 | 4.4 ± 0.4 | 4.8 ± 0.8 | 3.0 ± 0.6 | 4.6 ± 0.5 | 4.3 ± 0.4 |
| ***2. Hematologic data*** | | | | | | | | |
| **Heterophils (%)** | 52.8 ± 4.5 | 49.0 ± 3.6 | 63.9 ± 2.7 | 71.8 ± 3.7 | 54.5 ± 3.0 | 75.0 ± 4.3  (n=5) | 56.1 ± 1.9 | 68.3 ± 2.4  (n=29) |
| **Lymphocytes (%)** | 38.8 ± 4.3 | 49.4 ± 3.9 | 34.6 ± 2.7 | 26.3 ± 3.5 | 40.9 ± 3.2 | 23.0 ± 4.4  (n=5) | 41.2 ± 1.8 | 29.4 ± 2.5  (n=29) |
| **Monocytes (%)** | 4.5 ± 0.9 | 1.5 ± 0.4 | 1.3 ± 0.2 | 1.5 ± 0.3 | 3.8 ± 1.2 | 2.0 ± 0.8  (n=5) | 2.1 ± 0.3 | 1.3 ± 0.2  (n=29) |
| **Eosinophils (%)** | 4.0 ± 1.9 | 0.1 ± 0.1 | 0.3 ± 0.1 | 0.4 ± 0.2 | 0.9 ± 0.6 | 0.0 ± 0.0  (n=5) | 0.5 ± 0.2 | 0.3 ± 0.1  (n=29) |
| **Heterophils** (cells/uL) | 3680 ± 477 | 2517 ± 194 | 3784 ± 458 | 5045 ± 1110 | 3460 ± 395 | 5656 ± 1018  (n=5) | 3585 ± 284 | 7066 ± 1127  (n=29) |
| **Lymphocytes** (cells/uL) | 2783 ± 462 | 2631 ± 345 | 1873 ± 142 | 1386 ± 126 | 2513 ± 241 | 1558 ± 232  (n=5) | 2548 ± 180 | 2493 ± 199  (n=29) |
| **Monocytes** (cells/uL) | 327 ± 83 | 70 ± 15 | 75 ± 18 | 92 ± 17 | 253 ± 89 | 147 ± 59  (n=5) | 133 ± 22 | 209 ± 92  (n=29) |
| **Eosinophils** (cells/uL) | 272 ± 150 | 7 ± 7 | 14 ± 6 | 17 ± 10 | 49 ± 32 | 0 ± 0  (n=5) | 31 ± 10 | 34 ± 15  (n=29) |
| ***3. Timing data*** | | | | | | | | |
| **Bleed time** (min) | 2.63 ± 1.27 | 3.58 ± 3.08 | 2.33 ± 1.39 | 1.36 ± 0.68 | 2.18 ± 0.46 | 2.50 ± 1.21 | 1.84 ± 0.43 | 1.22 ± 0.61 |
| **Handling time**  (min) | 8.14 ± 1.70  (n=7) | 7.21 ± 3.59  (n=4) | (not recorded) | (not recorded) | 10.18 ± 0.69  (n=5) | 6.67 ± 1.56 | 5.97 ± 1.08  (n=29) | 4.85 ± 1.37  (n=29) |
| **CG4 lag time** (min) | 1.87 ± 0.35  (n=7) | 1.75 ± 1.05 | 2.19 ± 1.01 | 2.52 ± 1.51 | 1.60 ± 0.31 | 1.46 ± 0.84 | 1.97 ± 1.50 | 1.60 ± 0.53 |
| **CG8 lag time**  (min) | 6.20 ± 0.48 | 7.55 ± 3.65 | 5.69 ± 0.84 | 6.07 ± 1.52 | 5.29 ± 0.36 | 5.09 ± 0.82 | 5.60 ± 1.14 | 5.23 ± 0.61 |
