# Supplementary material for: Effects of Ground Transport in Kemp’s Ridley (Lepidochelys kempii) and Loggerhead (Caretta caretta) Turtles
Source: Integr Org Biol. 2020 May 19;2(1):obaa012. doi: 10.1093/iob/obaa012 (PMC7671109; doi:10.1093/iob/obaa012)
Supplement: obaa012_Supplementary_Data [file obaa012_supplementary_data.zip › Table S7.docx]

**Table S7.** Loggerhead transport-event data for vital rates, hematology and timing. Number of turtles studied per duration is shown at top; exceptions with lower *n* (e.g., due to i-STAT cartridge failure, hemolysis, or shipping delays) are shown in the applicable cells. Mean ± SEMs (vital rates, hematologic data) or mean ± standard deviations (timing data) are shown in each cell.

|  | **LOGGERHEAD TURTLES - TRANSPORT DATA** | | | | | | | |
| --- | --- | --- | --- | --- | --- | --- | --- | --- |
|  | **<6 h** | | **~12 h** | | **~18 h** | | **~24 h** | |
|  | **Pre**  (*n*=8) | **Post**  (*n*=8) | **Pre**  *(n*=10) | **Post**  (*n*=10) | **Pre**  (*n*=8) | **Post**  (*n*=8) | **Pre**  (*n*=8) | **Post**  (*n*=8) |
| ***1. Vital rates*** | | | | | | | | |
| **Cloacal Temp.** (°C) | 24.4 ± 0.2 | 24.4 ± 0.4 | 25.1 ± 0.7 | 25.5 ± 0.6 | 22.8 ± 0.3 | 24.3 ± 0.6 | 22.9 ± 0.5 | 23.3 ± 0.4 |
| **Heart Rate**  (bpm) | 29.0 ± 2.9 | 20.5 ± 3.8 | 36.5 ± 2.2 | 33.4 ± 4.5 | 30.6 ± 1.1 | 32.5 ± 1.1 | 31.6 ± 2.1 | 32.3 ± 1.7 |
| **Respiration**  (per min) | 3.1 ± 0.8 | 1.9 ± 0.7 | 3.2 ± 0.4 | 2.6 ± 0.8 | 3.5 ± 0.7 | 3.4 ± 0.7 | 2.6 ± 0.6 | 3.3 ± 0.8 |
| ***2. Hematologic data*** | | | | | | | | |
| **Heterophils (%)** | 49.1 ± 6.2 | 49.0 ± 7.4 | 58.2 ± 3.7 | 75.4 ± 5.4 | 54.5 ± 4.0 | 89.4 ± 2.2  (n=5) | 66.6 ± 3.4  (n=7) | 82.6 ± 2.1 |
| **Lymphocytes (%)** | 38.5 ± 6.7 | 38.9 ± 7.2 | 31.4 ± 3.3 | 21.4 ± 5.0 | 40.4 ± 4.2 | 7.2 ± 1.8  (n=5) | 27.1 ± 2.3  (n=7) | 14.1 ± 2.3 |
| **Monocytes (%)** | 5.1 ± 1.0 | 5.4 ± 0.9 | 2.9 ± 0.7 | 1.1 ± 0.3 | 2.5 ± 0.5 | 3.4 ± 1.0  (n=5) | 3.1 ± 0.6  (n=7) | 2.9 ± 0.7 |
| **Eosinophils (%)** | 7.3 ± 1.8 | 6.6 ± 1.5 | 7.5 ± 1.8 | 2.0 ± 0.8 | 2.6 ± 0.6 | 0.0 ± 0.0  (n=5) | 3.1 ± 1.2  (n=7) | 0.4 ± 0.3 |
| **Heterophils** (cells/uL) | 3158 ± 491 | 3541 ± 833 | 4611 ± 437 | 10868 ± 2317 | 1678 ± 25  (n=3) | 32872 ± 8410  (n=5) | 4137 ± 650  (n=3) | 22290 ± 5833 |
| **Lymphocytes** (cells/uL) | 2853 ± 795 | 2981 ± 721 | 2440 ± 273 | 2447 ± 569 | 2102 ± 270  (n=3) | 2104 ± 340  (n=5) | 1353 ± 264  (n=3) | 2797 ± 404 |
| **Monocytes** (cells/uL) | 312 ± 57 | 351 ± 47 | 212 ± 46 | 144 ± 49 | 41 ± 23  (n=3) | 1244 ± 430  (n=5) | 186 ± 59  (n=3) | 874 ± 338 |
| **Eosinophils** (cells/uL) | 427 ± 102 | 454 ± 112 | 557 ± 132 | 179 ± 60 | 113 ± 38  (n=3) | 0 ± 0  (n=5) | 191 ± 59  (n=3) | 52 ± 34 |
| ***3. Timing data*** | | | | | | | | |
| **Bleed time** (min) | 5.17 ± 3.2  (n=7) | 2.57 ± 1.80 | 2.26 ± 0.45 | 3.75 ± 3.52 | 2.62 ± 0.79 | 1.99 ± 0.82 | 3.14 ± 0.74 | 2.98 ± 2.29 |
| **Handling time**  (min) | 9.97 ± 2.07 | 11.74 ± 1.71  (n=6) | 6.78 ± 0.87  (n=4) | 8.01 ± 2.12  (n=4) | 5.62 ± 1.11  (n=5) | 7.89 ± 2.92 | 8.61 ± 2.20 | 6.11 ± 0.43  (n=5) |
| **CG4 lag time** (min) | 1.40 ± 0.31 | 1.36 ± 0.35 | 2.49 ± 1.63 | 1.45 ± 0.43 | 1.51 ± 0.72 | 1.17 ± 0.23 | 1.22 ± 0.32 | 1.39 ± 0.44 |
| **CG8 lag time**  (min) | 5.91 ± 1.24 | 5.04 ± 2.65 | 6.80 ± 2.04 | 5.48 ± 0.72 | 5.36 ± 1.03 | 5.01 ± 0.27 | 5.06 ± 0.39 | 5.34 ± 0.33 |
